# Hierarchical heterostructures of Bi_2_MoO_6_ microflowers decorated with Ag_2_CO_3_ nanoparticles for efficient visible-light-driven photocatalytic removal of toxic pollutants

**DOI:** 10.3762/bjnano.9.214

**Published:** 2018-08-27

**Authors:** Shijie Li, Wei Jiang, Shiwei Hu, Yu Liu, Yanping Liu, Kaibing Xu, Jianshe Liu

**Affiliations:** 1 Key Laboratory of Key Technical Factors in Zhejiang Seafood Health Hazards Institute of Innovation & Application, Zhejiang Ocean University, Zhoushan, Zhejiang Province 316022, China; 2 Department of Environmental Engineering, Zhejiang Ocean University, Zhoushan, Zhejiang Province 316022, China; 3 State Key Laboratory for Modification of Chemical Fibers and Polymer Materials, Research Center for Analysis and Measurement, Donghua University, Shanghai 201620, China; 4 State Environmental Protection Engineering Center for Pollution Treatment and Control in Textile Industry, College of Environmental Science and Engineering, Donghua University, Shanghai 201620, China

**Keywords:** antibiotic removal, Bi_2_MoO_6_, heterojunction, silver carbonate (Ag_2_CO_3_)

## Abstract

Developing highly active and durable visible-light-driven photocatalysts for the degradation of toxic pollutants is of vital significance. Herein, Ag_2_CO_3_ nanoparticles were in situ formed on Bi_2_MoO_6_ microflowers to produce Ag_2_CO_3_/Bi_2_MoO_6_ heterostructures via a facile procedure. The morphologies, phases, chemical compositions, and optical properties of Ag_2_CO_3_/Bi_2_MoO_6_ were examined by multiple characterization techniques. The Ag_2_CO_3_/Bi_2_MoO_6_ heterostructures exhibited substantially improved performance in the removal of industrial dyes (rhodamine B (RhB), methyl orange (MO), and methyl blue (MB)), and the antibiotic tetracycline hydrochloride (TC), compared with bare Bi_2_MoO_6_ and Ag_2_CO_3_ under visible-light irradiation. The enhancement of activity was attributed to the high charge-separation capacity, which results from the matched band alignment of the two components. The cycling experiments showed a good durability of Ag_2_CO_3_/Bi_2_MoO_6_. Holes were found to be the dominant active species accounting for the pollutant degradation. This compound is a promising candidate for wastewater treatment.

## Introduction

Industrial pollutants, such as industrial dyes and antibiotics, in wastewaters pose a huge threat to the environment [1–2]. Thus, many methods for pollutant removal have been established. However, the conventional wastewater treatments are usually accompanied by high cost, low efficiency and other insufficiencies [2]. The decomposition and mineralization of pollutants under sunlight through photocatalysis has been demonstrated to be an effective and green technology for environmental remediation [3–6]. Crucial to photocatalysis is to obtain high-performance photocatalysts [7–8]. Obtaining excellent photocatalysts that can be excited by visible light (43% of the solar energy spectrum) is very important for practical applications [9–14].

Bi_2_MoO_6_ has been regarded as a promising visible-light-driven (VLD) photocatalyst because of its good activity, chemical stability and nontoxicity [15–17]. However, the low carrier-separation rate and narrow photo-response range of Bi_2_MoO_6_ substantially lower its photocatalytic performance [18–19]. To overcome this obstacle, various methods have been developed, including doping [20–21] and the construction of heterojunctions [22–33]. Particularly, the combination of Bi_2_MoO_6_ with other semiconductors to construct heterojunction photocatalysts leads to an enhanced activity of Bi_2_MoO_6_, which originates from the increased charge separation at the interface [22–30].

Recently, Ag-based compounds (e.g., Ag_3_PO_4_, Ag_3_VO_4_, Ag_2_CrO_4_, and Ag_2_CO_3_) [34–36] have emerged as good VLD photocatalysts for pollutant removal. Ag_2_CO_3_ exhibits a high visible-light photocatalytic activity [37]. However, it is unstable under illumination. Previous studies found that the good stability of Ag_2_CO_3_ could be achieved through the rational construction of heterojunctions, such as Ag_2_CO_3_/Bi_2_WO_6_ [37], Ag_2_O/Ag_2_CO_3_ [38], Ag_2_CO_3_/Bi_2_O_2_CO_3_ [39], Ag_2_CO_3_/C_3_N_4_ [40], Ag/Ag_2_CO_3_/BiVO_4_ [41] Ag_2_CO_3_/AgBr/ZnO [42], and Ag/Ag_2_CO_3_/Bi2MoO_6_ [32]. The band structure of Ag_2_CO_3_ matches well with that of Bi_2_MoO_6_ [32]. Moreover, morphology modulation is another significant way to enhance photocatalytic activity. Three-dimensional nanostructures endow materials with unique physicochemical properties, for instance, high specific surface area, good molecular diffusion/transport, and good recyclability and light harvesting ability. To the best of our knowledge, application of Ag_2_CO_3_ nanoparticles coupled with flower-like Bi_2_MoO_6_ for photocatalytic degradation of toxic pollutants remains unreported.

Herein, we synthesized flower-like Ag_2_CO_3_/Bi_2_MoO_6_ heterostructures, in which Ag_2_CO_3_ nanoparticles were evenly anchored on Bi_2_MoO_6_ microflowers to construct novel hierarchical heterojunction photocatalysts by via in situ precipitation. The photocatalytic properties of Ag_2_CO_3_/Bi_2_MoO_6_ was measured regarding the photocatalytic degradation of industrial dyes (rhodamine B (RhB), methyl orange (MO), and methyl blue (MB)), and the antibiotic tetracycline hydrochloride (TC) under visible light. The improved performance of the photocatalytic degradation was prominent, and the reasons were rationally analyzed. Also, the photocatalytic mechanism of pollutant degradation over Ag_2_CO_3_/Bi_2_MoO_6_ was discussed.

## Results and Discussion

### Characterization of catalysts

**Figure 1:**
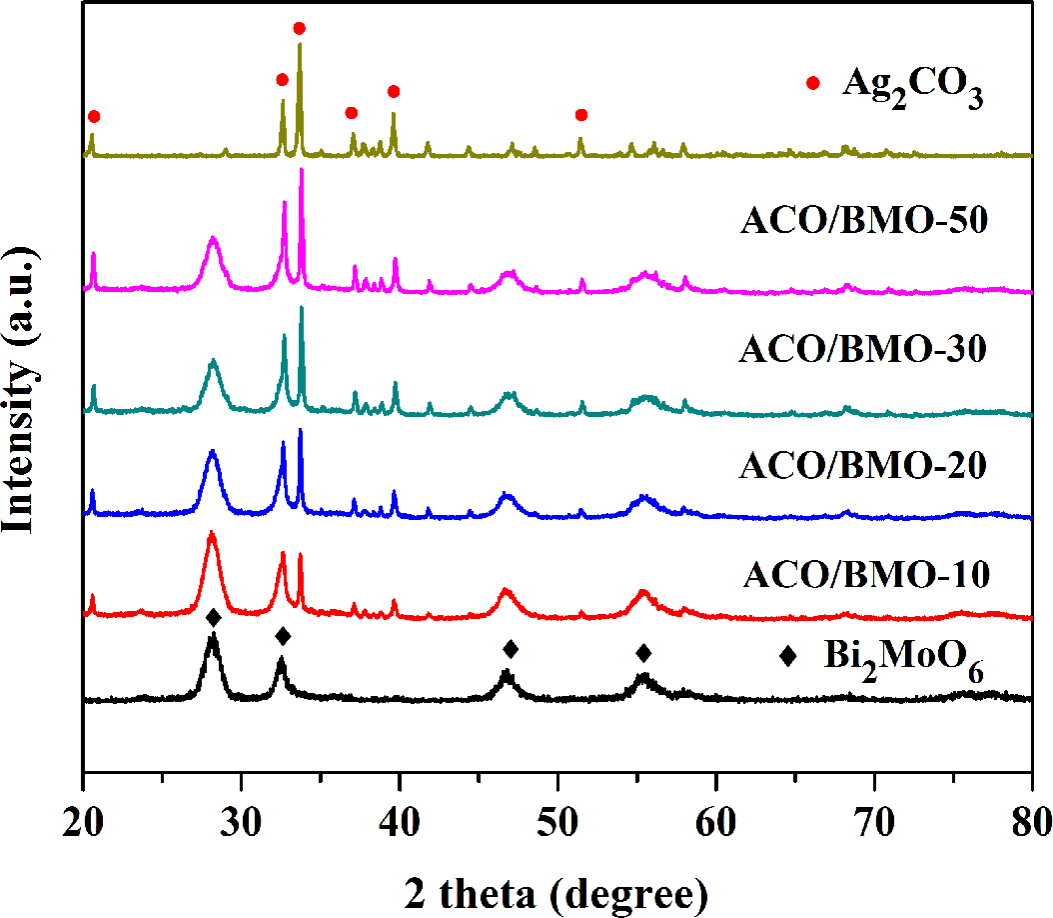
XRD patterns of Ag_2_CO_3_/Bi_2_MoO_6_ heterojunctions, bare Bi_2_MoO_6_ and Ag_2_CO_3_.

A series of flowerlike Ag_2_CO_3_/Bi_2_MoO_6_ heterostructures with different weight ratios (0.1/1, 0.2/1, 0.3/1, and 0.5/1) were constructed and labeled ACO/BMO-10, ACO/BMO-20, ACO/BMO-30, and ACO/BMO-50, respectively. The crystal structure of Bi_2_MoO_6_, Ag_2_CO_3_, and Ag_2_CO_3_/Bi_2_MoO_6_ heterostructures were determined by XRD technique (Figure 1). The diffraction peaks of Ag_2_CO_3_ and Bi_2_MoO_6_ were indexed as orthorhombic Bi_2_MoO_6_ (JCPDS 76-2388) and monoclinic Ag_2_CO_3_ (JCPDS 26-0399), respectively. The XRD pattern of these heterostructures show the characteristic peaks of both Ag_2_CO_3_ and Bi_2_MoO_6_, indicating the successful synthesis of Ag_2_CO_3_/Bi_2_MoO_6_ heterostructures. In another publication, Ag/Ag_2_CO_3_/Bi_2_MoO_6_ nanoplates, composed of three phases, have been described [32].

**Figure 2:**
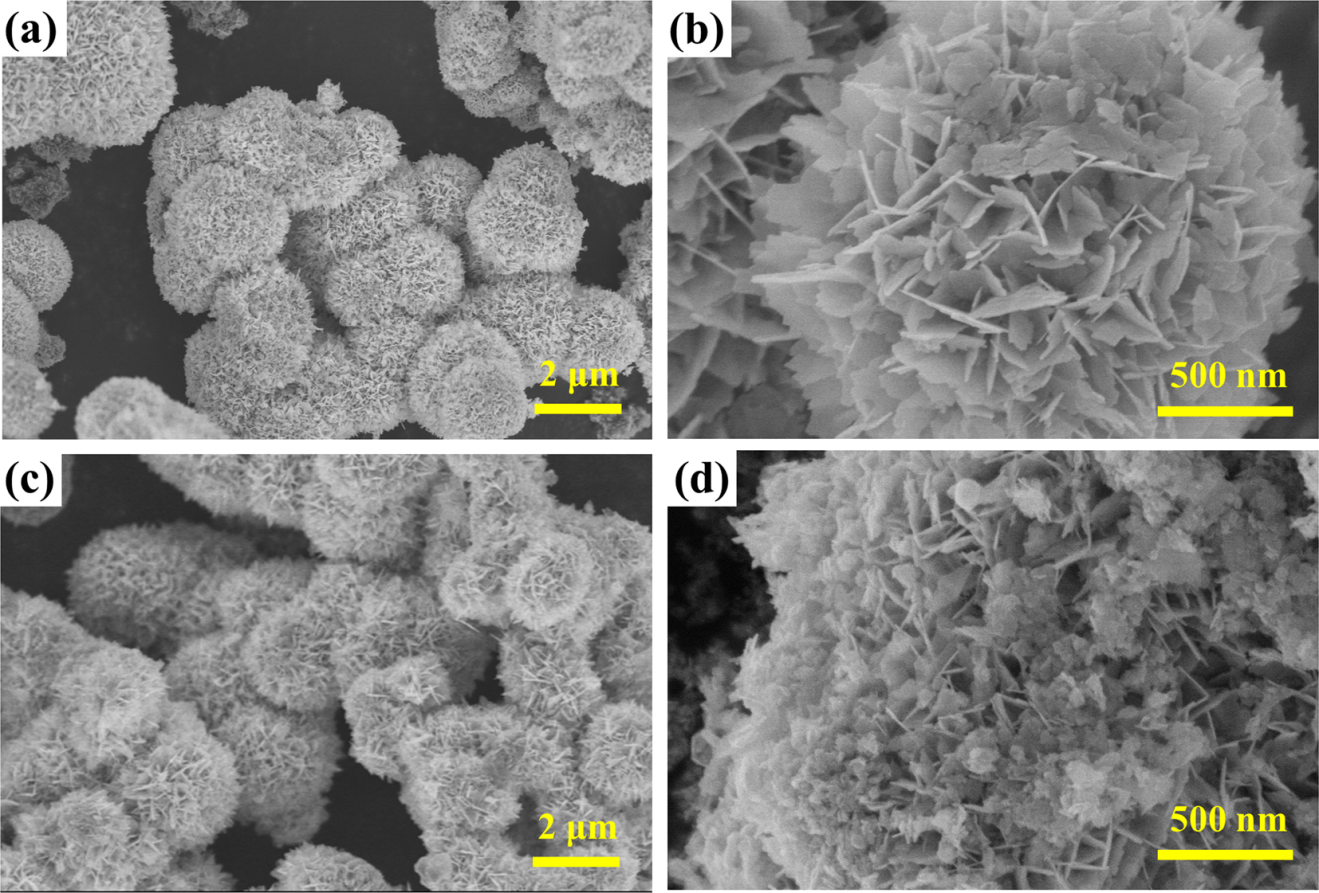
SEM images of (a, b) bare Bi_2_MoO_6_ and (c, d) ACO/BMO-30.

To visually study the microstructure and morphology of Ag_2_CO_3_/Bi_2_MoO_6_, SEM images of the as-prepared catalysts were taken. Bare Bi_2_MoO_6_ presents a hierarchical microsphere structure (diameter: 1.6–3.5 μm, Figure 2a,b). After Ag_2_CO_3_ was loaded onto Bi_2_MoO_6_, the resulting Ag_2_CO_3_/Bi_2_MoO_6_ retained the flower-like architecture. The representative ACO/BMO-30 displays the flower-like structure, the surface of which is decorated with Ag_2_CO_3_ nanoparticles (size: 10–50 nm, Figure 2c,d). In contrast, the previously reported Ag/Ag_2_CO_3_/Bi_2_MoO_6_ is composed of Bi_2_MoO_6_ nanoplates and Ag/Ag_2_CO_3_ nanorods/nanoparticles [32].

**Figure 3:**
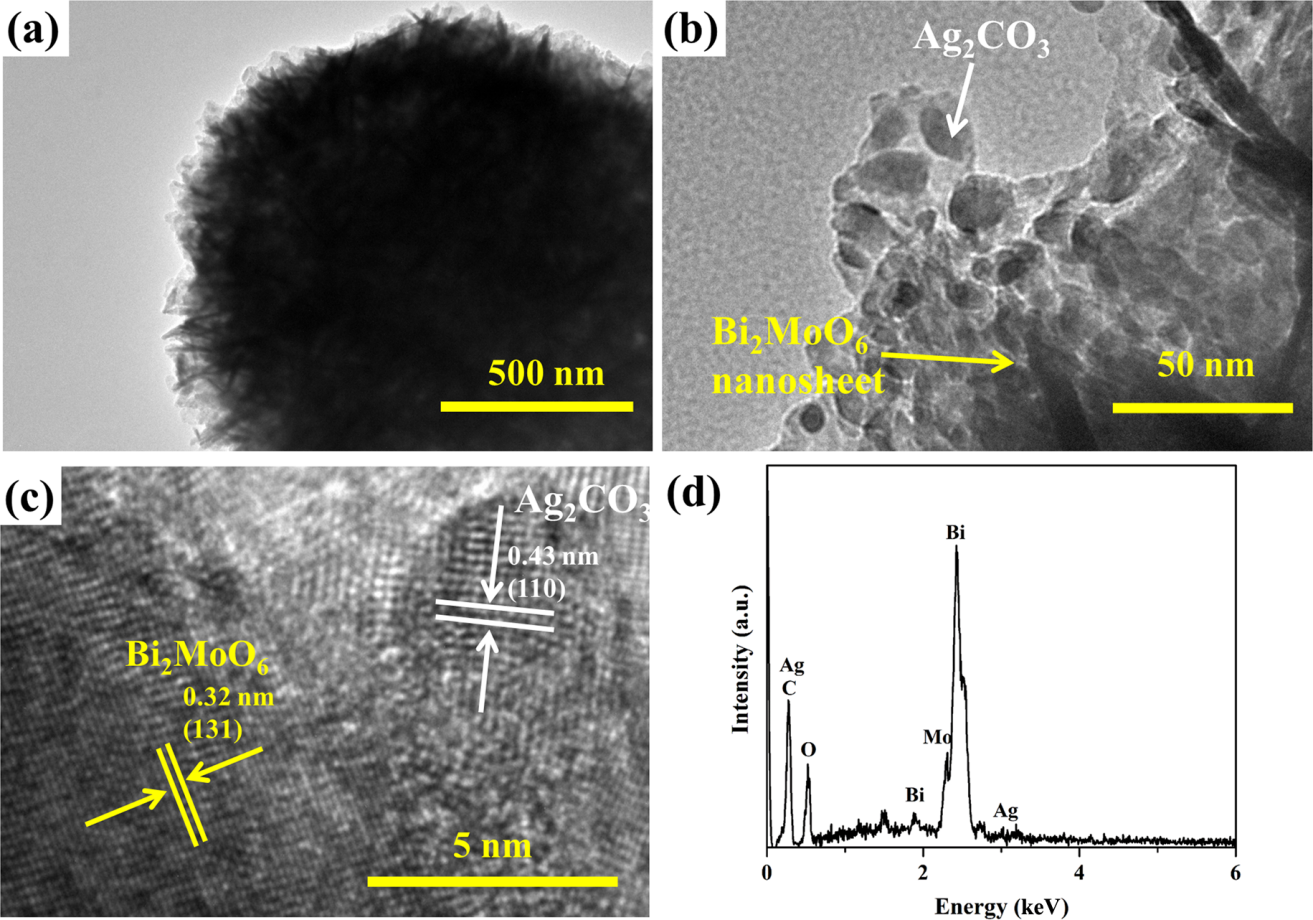
(a–c) TEM images of ACO/BMO-30; (d) EDS pattern of ACO/BMO-30.

Further information about the structure of ACO/BMO-30 was collected from TEM images (Figure 3). The TEM images are in line with the SEM observations, i.e., ACO/BMO-30 exhibits a flower-like architecture loaded with Ag_2_CO_3_ nanoparticles (Figure 3a,b). The HRTEM displays two different lattice spacings of 0.32 and 0.43 nm, which match well with the (121) planes of orthorhombic Bi_2_MoO_6_ and the (110) planes of monoclinic Ag_2_CO_3_ (Figure 3c). Moreover, the energy-dispersive spectroscopy (EDS) pattern confirmed the existence of Ag, C, O, Bi, and Mo in ACO/BMO-30 (Figure 3d).

**Figure 4:**
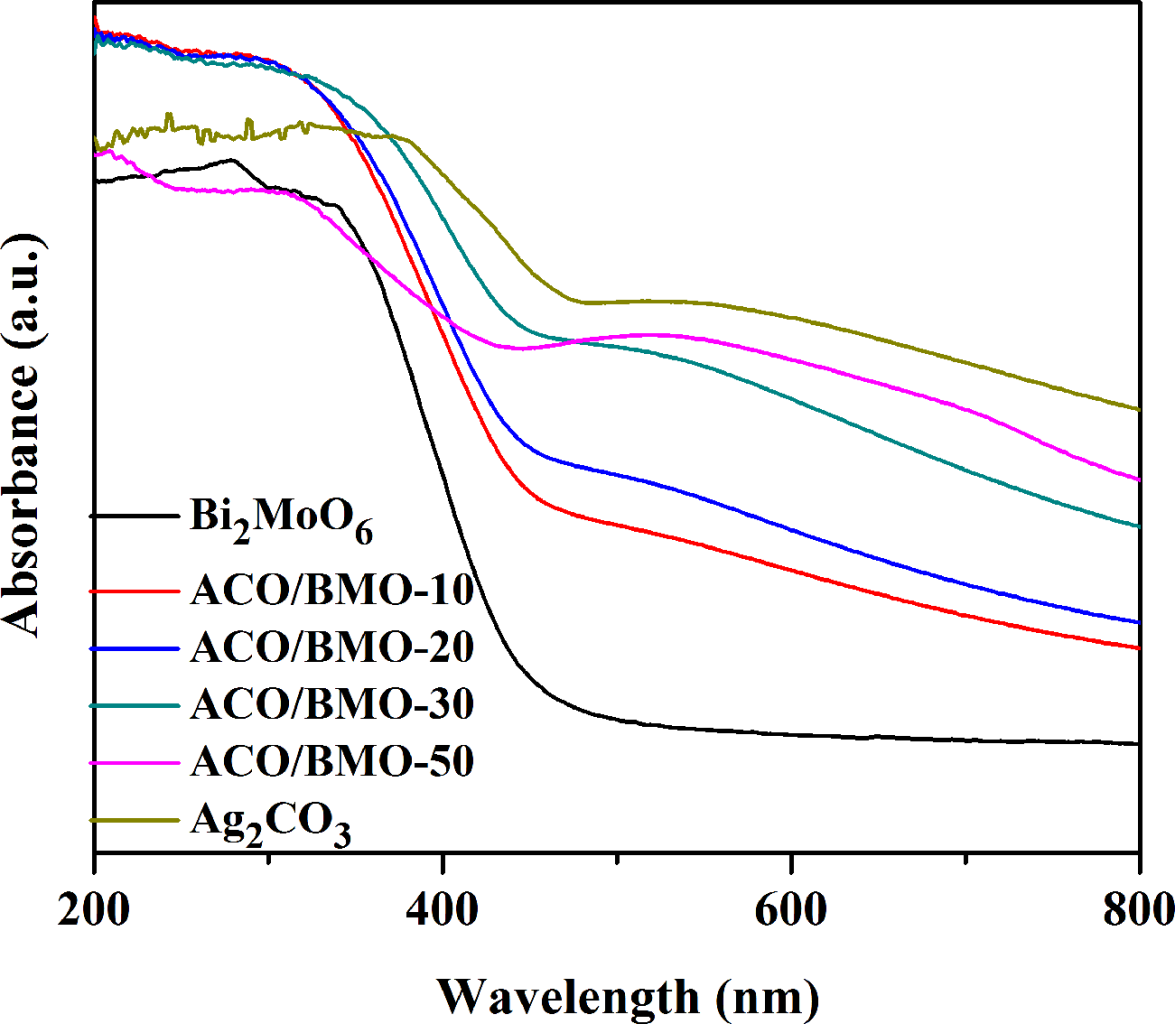
UV–vis diffuse reflection spectra of bare Bi_2_MoO_6_, Ag_2_CO_3_ and Ag_2_CO_3_/Bi_2_MoO_6_ heterostructures.

The optical absorption of Bi_2_MoO_6_, Ag_2_CO_3_ and Ag_2_CO_3_/Bi_2_MoO_6_ heterostructures were measured by using UV–vis diffuse reflection spectra (UV–vis DRS, Figure 4). The absorption edges of Ag_2_CO_3_ and Bi_2_MoO_6_ are around 570 nm and 470 nm, respectively, in line with already reported values [30,33,39]. Compared to pristine Bi_2_MoO_6,_ the absorption of the Ag_2_CO_3_/Bi_2_MoO_6_ heterostructures was substantially improved owing through the introduction of Ag_2_CO_3_ nanoparticles. Ag/Ag_2_CO_3_/Bi_2_MoO_6_ [32], Ag_2_MoO_4_/Bi_2_MoO_6_ [22], and Ag_2_CO_3_/Bi_2_MoO_6_ heterostrutures are VLD photocatalysts.

The band gap energy (*E*_g_) can be estimated from the Tauc plot: (α*h*ν) *= A*(*h*ν − *E*_g_)*^n^*^/2^. Where α, *h*, ν, and *A* are absorption coefficient, Planck’s constant, the frequency of light, and a constant, respectively. The value of *n* depends on the type of electronic transition and *n* is equal to 1 for Bi_2_MoO_6_ and Ag_2_CO_3_. The Tauc plots of Ag_2_CO_3_ and Bi_2_MoO_6_ converted from the UV–vis DRS measurements are shown in Figure S1 (Supporting Information File 1). The band gaps are determined to be 2.17 for Ag_2_CO_3_ and 2.66 eV for Bi_2_MoO_6_.

The band potentials of Ag_2_CO_3_ and Bi_2_MoO_6_ can be estimated by the empirical equations:

[1]
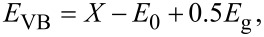


[2]
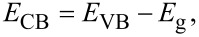


where the *X* value for Bi_2_MoO_6_ is 5.5 eV [43], and that for Ag_2_CO_3_ is 6.02 eV [44]. The value of *E*_0_ is ca. 4.5 eV. Hence, the values of *E*_VB_ and *E*_CB_ of Bi_2_MoO_6_ were calculated as −0.32 and 2.34 eV, and those of Ag_2_CO_3_ were calculated as 0.43 and 2.60 eV.

**Figure 5:**
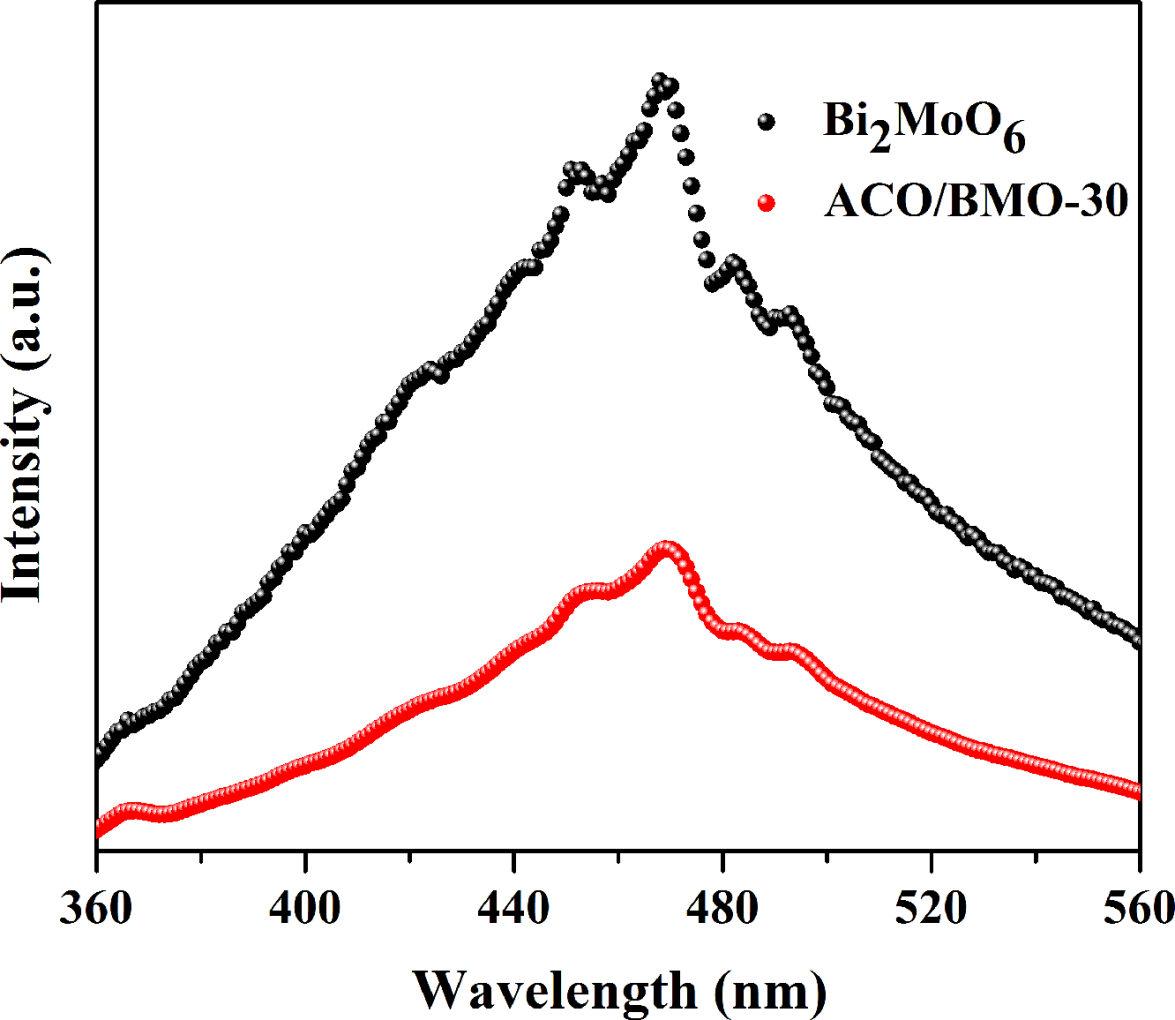
PL spectra of bare Bi_2_MoO_6_ and ACO/BMO-30.

PL spectra were measured to analyze the electron–hole-separation efficiency [45–47]. Figure 5 shows the contrast between the PL spectra of bare Bi_2_MoO_6_ and of ACO/BMO-30. The emission peak of Bi_2_MoO_6_ is centered at ca. 465 nm under an excitation wavelength of 300 nm. Intriguingly, the PL emission intensity of Bi_2_MoO_6_ was reduced after the introduction of Ag_2_CO_3_. A similar phenomenon was also found in Ag/Ag_2_CO_3_/Bi_2_MoO_6_ [32] and Ag_2_MoO_4_/Bi_2_MoO_6_ [22]. This result shows that the charge separation efficiency is enhanced in ACO/BMO-30.

### Photocatalytic performance

**Figure 6:**
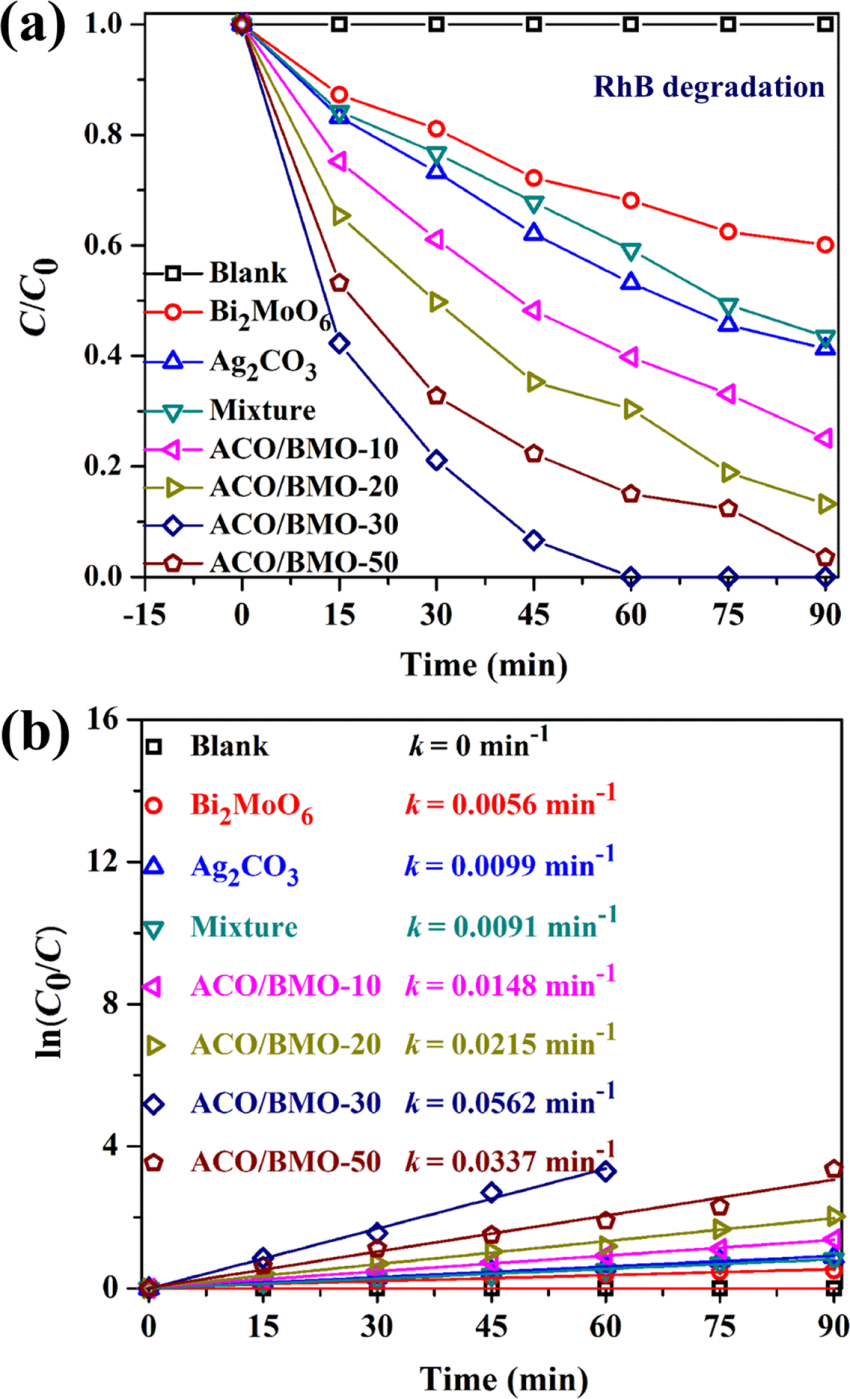
(a) Photocatalytic degradation of RhB over different samples under visible light. (b) Rate constants of RhB degradation for different catalysts.

The efficiency of Ag_2_CO_3_/Bi_2_MoO_6_ heterostructures in the photocatalytic degradation of industrial dyes (RhB, MO and MB), and the antibiotic TC of the under visible light was measured. Figure 6a displays the degradation of RhB as a function of the time. The RhB concentration remains unchanged in the absence of catalysts. In the presence of bare Bi_2_MoO_6_ and Ag_2_CO_3_ only 39.8% and 58.7% of RhB were degraded after 90 min of reaction time. The degradation of RhB was substantially enhanced when a combination of Bi_2_MoO_6_ and Ag_2_CO_3_ was used. For instance, the introduction of a low amount of Ag_2_CO_3_ (10 wt %) resulted in 74.9% degradation of RhB. Obviously, the RhB degradation performance is closely related to the loading amount of Ag_2_CO_3_. Among the heterostructures, ACO/BMO-30 achieves the best activity in the degradation of RhB, with 100% degradation efficiency after 60 min of reaction time. ACO/BMO-10, ACO/BMO-20 and ACO/BMO-50 showed degradation efficiencies of 60.2%, 69.6%, and 85.1%, respectively. Remarkably, a mixture of 23.1 wt % Ag_2_CO_3_ and 76.9 wt % Bi_2_MoO_6_ exhibited a much lower activity than ACO/BMO-30, verifying that the close contact between the components also has a significant influence on the photocatalytic performance of the heterostructures.

The pseudo-first-order kinetic plots and rate constants of RhB degradation for various catalysts are presented in Figure 6b. The degradation rate constant of ACO/BMO-30 is 0.0562 min^−1^, which 9.0-, 4.7- and 5.2-fold higher than that of bare Bi_2_MoO_6_ (0.0056 min^−1^), Ag_2_CO_3_ (0.0099 min^−1^), and the mixture (0.0091 min^−1^).

**Figure 7:**
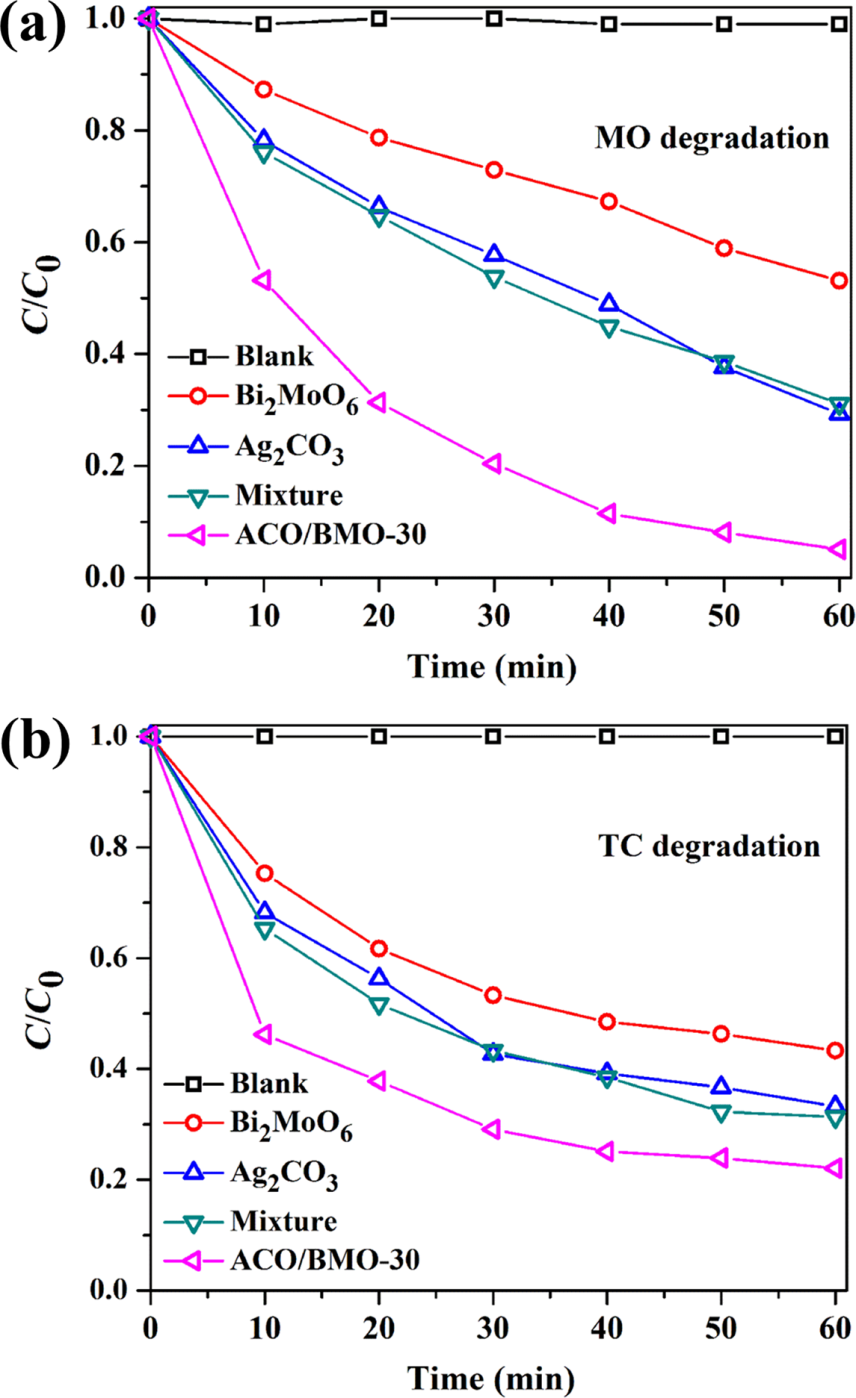
Photocatalytic degradation of (a) MO and (b) TC over different samples under visible light.

Figure 7 and Figure S2 (Supporting Information File 1) show the degradation of MO and TC as a function of the time. Again, ACO/BMO-30 displayed the highest activity, with degradation efficiencies of 94.9% for MO, 100% for MB, and 78.9% for TC. The photocatalytic activity in the degradation of TC of ACO/BMO-30 was further compared with that of Ag/Ag_2_CO_3_/Bi_2_MoO_6_ [32], and of Ag_2_MoO_4_/Bi_2_MoO_6_ [22]. As shown in Figure S3 (Supporting Information File 1), ACO/BMO-30 is much more active than Ag_2_MoO_4_/Bi_2_MoO_6_, but not as active as Ag/Ag_2_CO_3_/Bi_2_MoO_6_ due to the fact that Ag/Ag_2_CO_3_/Bi_2_MoO_6_ is a ternary composite.

To examine the mineralization ability of ACO/BMO-30, the total organic carbon (TOC) was measured during the RhB degradation, and the result is presented in Figure S4 (Supporting Information File 1). Apparently, the TOC removal efficiency gradually goes up with the increase of reaction time and the final TOC removal efficiency is as high as 80.6% after 5 h of reaction. Hence, ACO/BMO-30 shows a decent mineralization ability.

**Figure 8:**
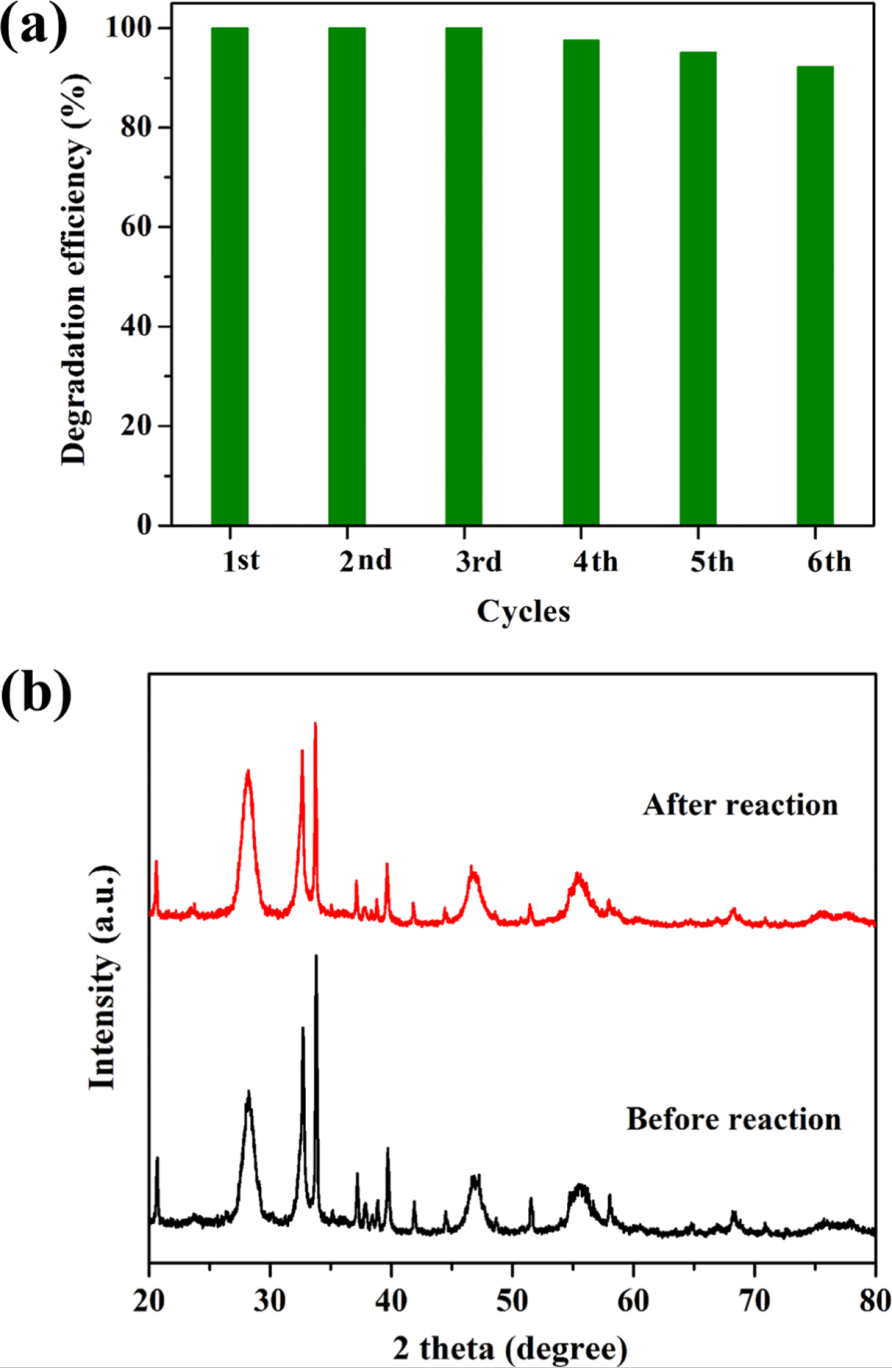
(a) The cycling performance of ACO/BMO-30. (b) The XRD patterns of ACO/BMO-30 before and after five reaction cycles.

To test the durability of ACO/BMO-30, repeated runs were carried out under unchanging conditions. As depicted in Figure 8a, after six consecutive runs, the RhB degradation efficiency is still about 92.2%. Additionally, no apparent change of the crystalline structure was found after the photocatalytic reactions, as shown in the XRD pattern (Figure 8b). However, the XPS pattern of the used ACO/BMO-30 suggests that some Ag^+^ is reduced to Ag(0) after the reaction (Figure S5, Supporting Information File 1). It has been recognized that the formation of Ag/Ag_2_CO_3_ shows a stable structure [32]. These results verify that ACO/BMO-30 is stable. It is rationally speculated that ACO/BMO-30 has great potential for the elimination of toxic pollutants.

### Photocatalytic mechanism

**Figure 9:**
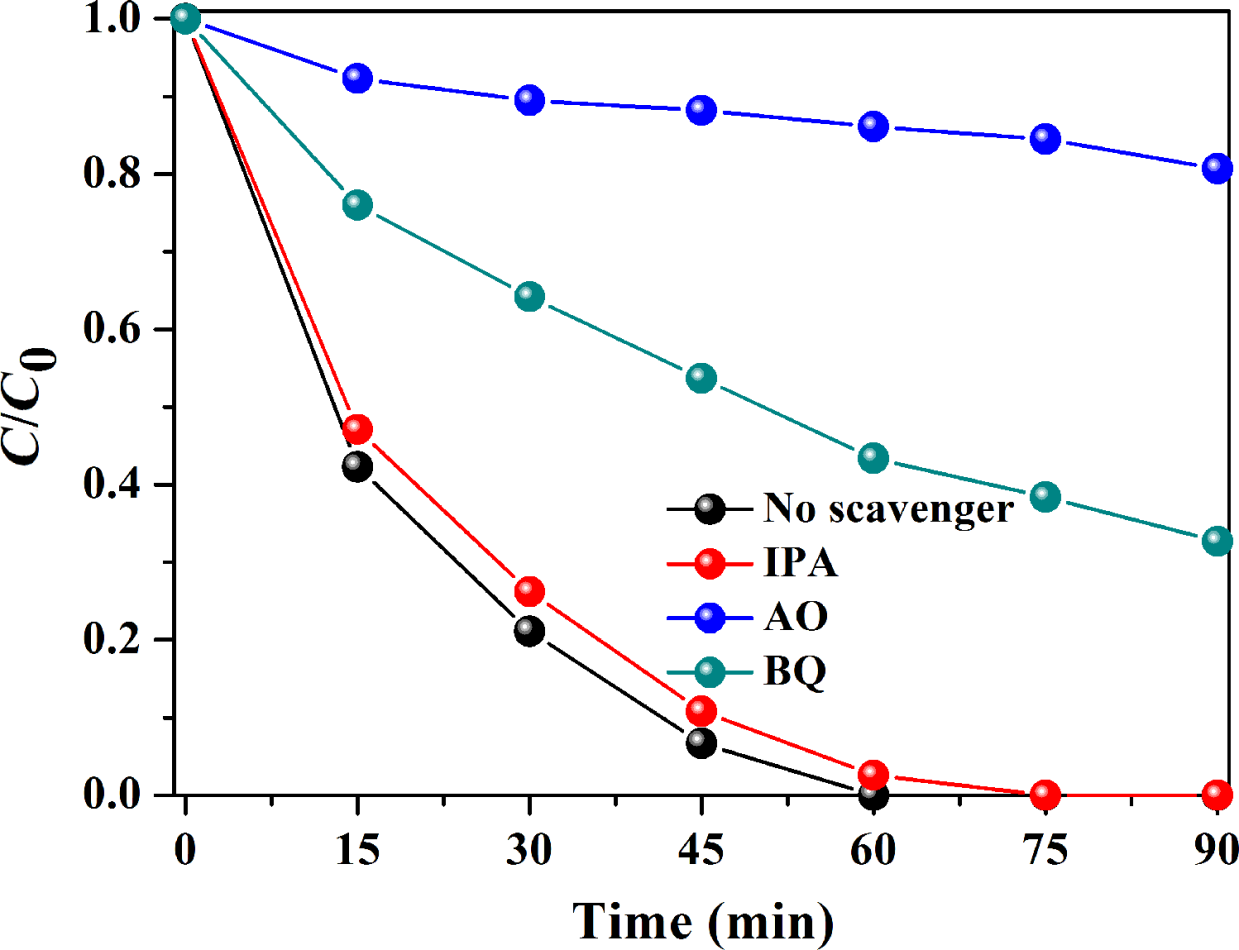
Radical-scavenger tests over ACO/BMO-30.

In general, radicals and other active species (e.g., •O_2_^−^, •OH and h^+^) produced in the photocatalytic reaction system contribute to the decomposition of pollutant [48–52]. We performed radical-scavenger tests to determine the active species in the degradation of RhB. As displayed in Figure 9, the addition of isopropanol (IPA) resulted in a slight inhibition of the RhB degradation, signifying that •OH is not the major active species. The addition of *p*-benzoquinone (BQ) makes the RhB degradation efficiency decline from 100% to 67.3%, suggesting that •O_2_^−^ plays a minor role. After the addition of ammonium oxalate (AO) the RhB degradation efficiency decreased from 100% to 19.3%, indicating that h^+^ plays a dominant role in the degradation of RhB over Ag_2_CO_3_/Bi_2_MoO_6_.

**Figure 10:**
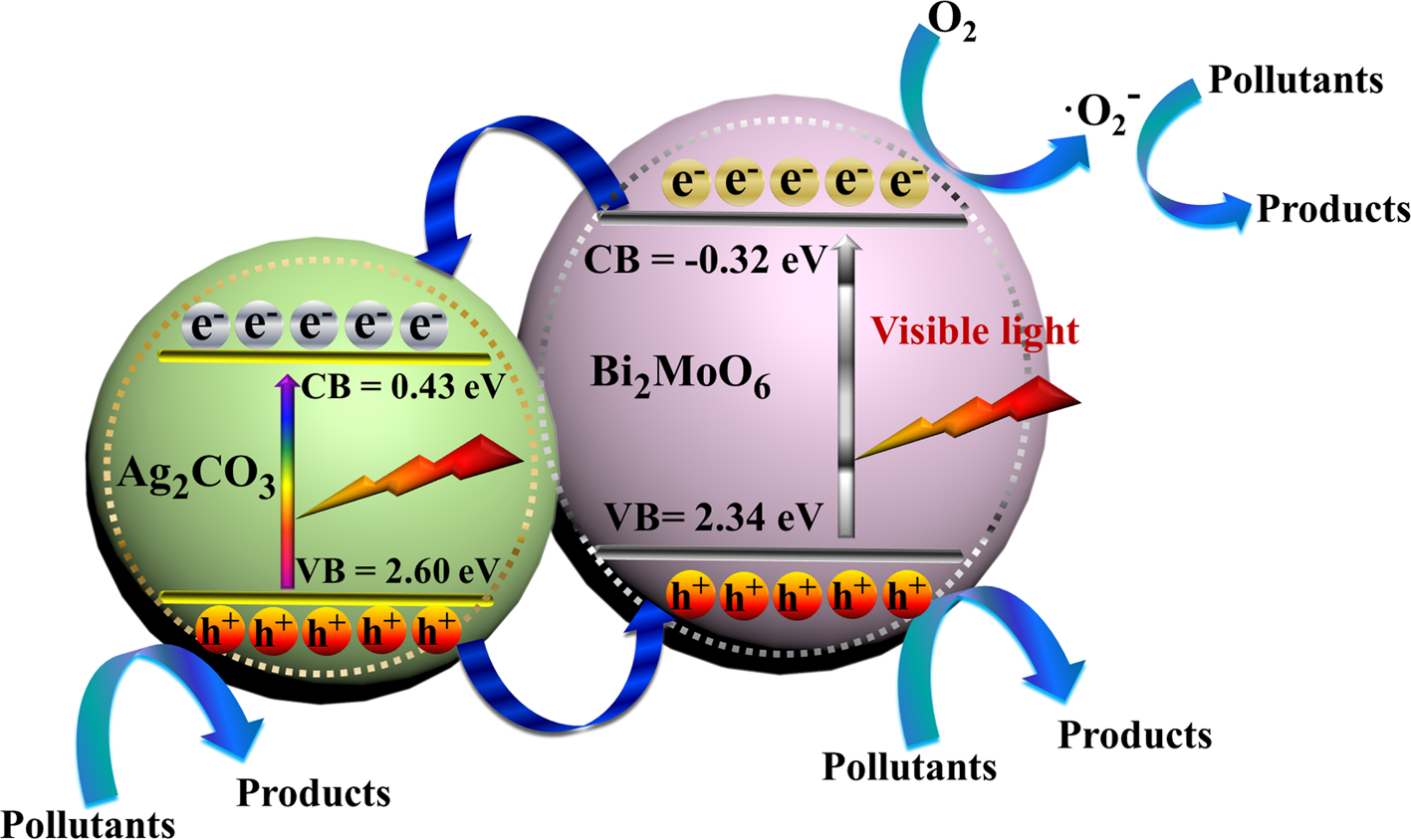
Proposed photocatalytic degradation mechanism of Ag_2_CO_3_/Bi_2_MoO_6_.

Based on the above results, a possible schematic mechanism is presented in Figure 10. In the Ag_2_MoO_4_/Bi_2_MoO_6_ system [22], electron–hole pairs can only be generated by visible light in Bi_2_MoO_6_. In comparison, both Ag_2_CO_3_ and Bi_2_MoO_6_ are able to absorb visible light and, and charge carriers are photo-generated on the surface of Ag_2_CO_3_ and Bi_2_MoO_6_. The electrons in the CB of Bi_2_MoO_6_ can partially transfer into that of Ag_2_CO_3_. The electrons are then captured by the oxygen, producing •O_2_^−^ radicals, which play a minor role in decomposing pollutants, as confirmed by the radical-scavenger test (Figure 9). At the same time, the holes in the VB of Ag_2_CO_3_ can partially flow into that of Bi_2_MoO_6._ The holes in both components mainly account for the pollutant degradation (Figure 9). Electron–hole recombination is severely inhibited, as verified by PL results (Figure 5) [53–54]. Although the VB potential of Ag_2_CO_3_ (2.6 eV) is more positive than that of H_2_O/•OH (2.38 eV/NHE at pH 7), the generated •OH radicals did not act as the major active species for pollutant degradation (Figure 9). In Ag/Ag_2_CO_3_/Bi_2_MoO_6_, Ag(0) can also be excited to generate electrons, reacting with O_2_ to form •O_2_^−^ radicals due to the plasmon resonance (SPR) effect [32]. Based on above analysis, the rational design and construction of Bi_2_MoO_6_-based heterostructures is favorable for the separation of charges, leading to a superior activity in pollutant degradation.

## Conclusion

We synthesized a novel photocatalyst of Bi_2_MoO_6_ microflowers covered with Ag_2_CO_3_ nanoparticles by a facile procedure. The Ag_2_CO_3_/Bi_2_MoO_6_ heterostructures, especially ACO/BMO-30, showed a substantially enhanced photocatalytic performance in the removal of pollutants (RhB, MO, MB and TC) under visible light compared to bare Bi_2_MoO_6_ and Ag_2_CO_3_. Moreover, ACO/BMO-30 possesses good durability and stability. The enhanced photocatalytic performance is ascribed to the extended optical response and the matched band structure, reducing carrier recombination. This study offers a novel highly efficient VLD photocatalyst with promising applications in environmental remediation.

## Experimental

### Materials

Bi(NO_3_)_3_·5H_2_O, NaHCO_3_, NH_3_·H_2_O, Na_2_MoO_4_·2H_2_O, rhodamine B (RhB), methyl blue (MB), tetracycline hydrochloride (TC), methyl orange (MO), isopropanol (IPA), AgNO_3_, *p*-benzoquinone (BQ), and ammonium oxalate (AO) were obtained from Chemical Reagent factory (China). All the reagents were used directly without further treatment.

### Synthesis

Flower-like Bi_2_MoO_6_ was synthesized using a solvothermal route. Typically, Na_2_MoO_4_·2H_2_O (1 mmol) was dissolved in a mixture of 40 mL of CH_3_CH_2_OH and 40 mL of ethylene glycol with the aid of ultrasonic treatment. Then Bi(NO_3_)_3_·5H_2_O (2 mmol) was also dissolved in the above solution in the same way. Subsequently, the solution was magnetically stirred for 1 h, and then loaded into a 100 mL Teflon container and reacted at 160 °C for 25 h. After the reaction system had cooled down to room temperature, the precipitants were washed thoroughly with deionized water and ethanol, dried, and calcined at 350 °C for 1 h.

Ag_2_CO_3_/Bi_2_MoO_6_ heterostructures were obtained by wet-chemical deposition. Typically, 0.6 g Bi_2_MoO_6_ was ultrasonically dispersed in 30 mL of deionized water. Then AgNO_3_ solution (0.1 M) was poured into the suspension and kept stirring for 1 h in the dark. Subsequently, NH_3_·H_2_O (0.05 M) was added into the above mixture. After that, NaHCO_3_ solution (0.1 M) was dropped into the above system slowly under magnetic stirring in the dark, followed by stirring for another 2 h. The collected solid was washed, and dried at 70 °C overnight. By varying the added amounts of AgNO_3_, NaHCO_3_, and NH_3_·H_2_O, the as-prepared Ag_2_CO_3_/Bi_2_MoO_6_ heterojunctions with various weight ratios (0. 1/1, 0.2/1, 0.3/1 and 0.5/1) were obtained and denoted as ACO/BMO-10, ACO/BMO-20, ACO/BMO-30, and ACO/BMO-50, respectively. For comparison, pure Ag_2_CO_3_ was prepared by the same method without the addition of Bi_2_MoO_6_. A mixture of Ag_2_CO_3_ and Bi_2_MoO_6_, symbolized as mixture, was prepared by simple physical mixing.

### Characterization

The microstructure of the samples was observed with a Hitachi S-4800 scanning electron microscope (SEM) and a JEM-2010F transmission electron microscope (TEM). The corresponding chemical compositions were detected by energy-dispersive X-ray (EDX) spectroscopy equipped on the SEM. The crystal structure of catalysts was identified by X-ray diffractometry (XRD, Bruker D8 Advance) with a scanning range of 2θ from 20° to 80°. The UV–vis spectra were recorded using an UV-2600 UV–vis spectrophotometer (Shimadzu). The photoluminescence (PL) emission spectra of the samples were recorded on a Hitachi F-7000 spectrophotometer, employing an excitation wavelength of 300 nm.

### Photocatalytic test

The photo-degradation of RhB, MO, TC and MB was carried out in a reactor containing 40 mg of sample, and 100 mL of RhB (10 mg·L^−1^), MO (10 mg·L^−1^), TC (20 mg·L^−1^) or MB (10 mg·L^−1^) under visible-light irradiation. A 300 W xenon lamp equipped with a UV-cutoff filter (λ > 400 nm) served as the light source. First, the suspension was stirred in the dark for half an hour. During reaction, 2 mL of solution was taken at certain intervals, and centrifuged to obtain the supernatant for UV–vis spectrophotometry measurements. Total organic carbon (TOC) tests were executed by the degradation of RhB (50 mg·L^−1^, 150 mL) solution with 300 mg of ACO/BMO-30 as the catalyst.

## Supporting Information

File 1 Additional experimental data.
