# Supplementary material for: Hierarchical heterostructures of Bi2MoO6 microflowers decorated with Ag2CO3 nanoparticles for efficient visible-light-driven photocatalytic removal of toxic pollutants
Source: Beilstein J Nanotechnol. 2018 Aug 27;9:2297–305. doi: 10.3762/bjnano.9.214 (PMC6122119; doi:10.3762/bjnano.9.214)
Supplement: File 1 — Additional experimental data. [file Beilstein_J_Nanotechnol-09-2297-s001.pdf]

Supporting Information

for

Hierarchical heterostructures of Bi_2MoO_6 microflowers decorated with Ag_2CO_3 nanoparticles for efficient visible-light-driven photocatalytic removal of toxic pollutants

Shijie Li*[1], Wei Jiang[1], Shiwei Hu[1], Yu Liu[1], Yanping Liu[2], Kaibing Xu*[3] and Jianshe Liu[4]

Address: [1]Key Laboratory of key technical factors in Zhejiang seafood health hazards Institute of Innovation & Application, Zhejiang Ocean University, Zhoushan, Zhejiang Province, 316022, China; [2]Department of Environmental Engineering, Zhejiang Ocean University, Zhoushan, Zhejiang Province, 316022, China; [3]State Key Laboratory for Modification of Chemical Fibers and Polymer Materials, Research Center for Analysis and Measurement, Donghua University, Shanghai, 201620, China and [4]State Environmental Protection Engineering Center for Pollution Treatment and Control in Textile Industry, College of Environmental Science and Engineering, Donghua University, Shanghai 201620, China

Email: Shijie Li - lishijie@zjou.edu.cn; Kaibing Xu- xukaibing@dhu.edu.cn

* Corresponding author

Additional experimental data

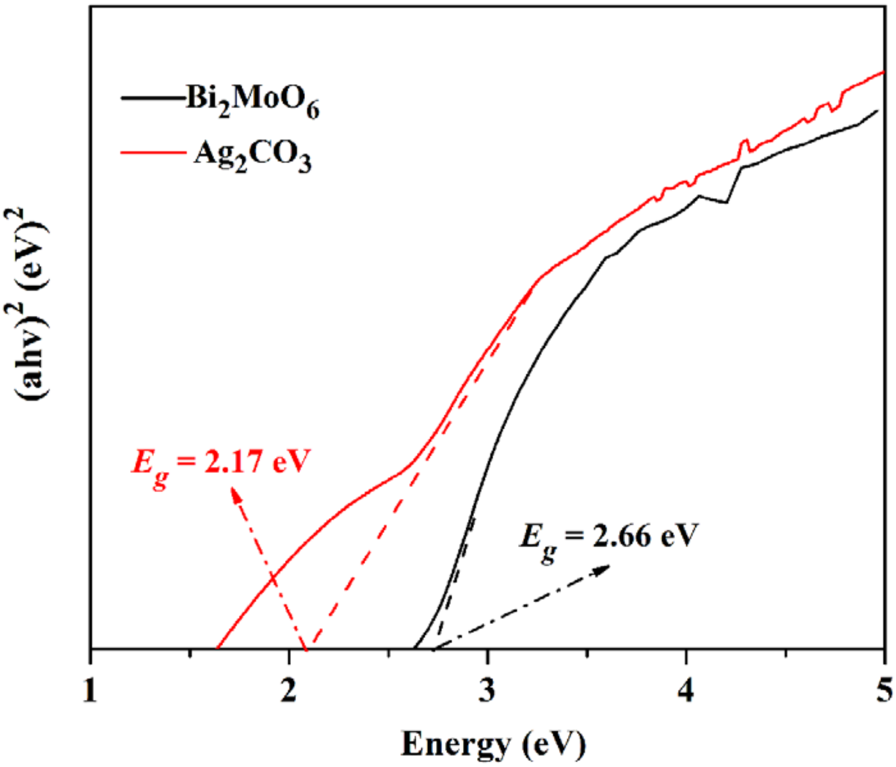

Figure S1: Tauc plots of Ag_2CO_3 and Bi_2MoO_6.

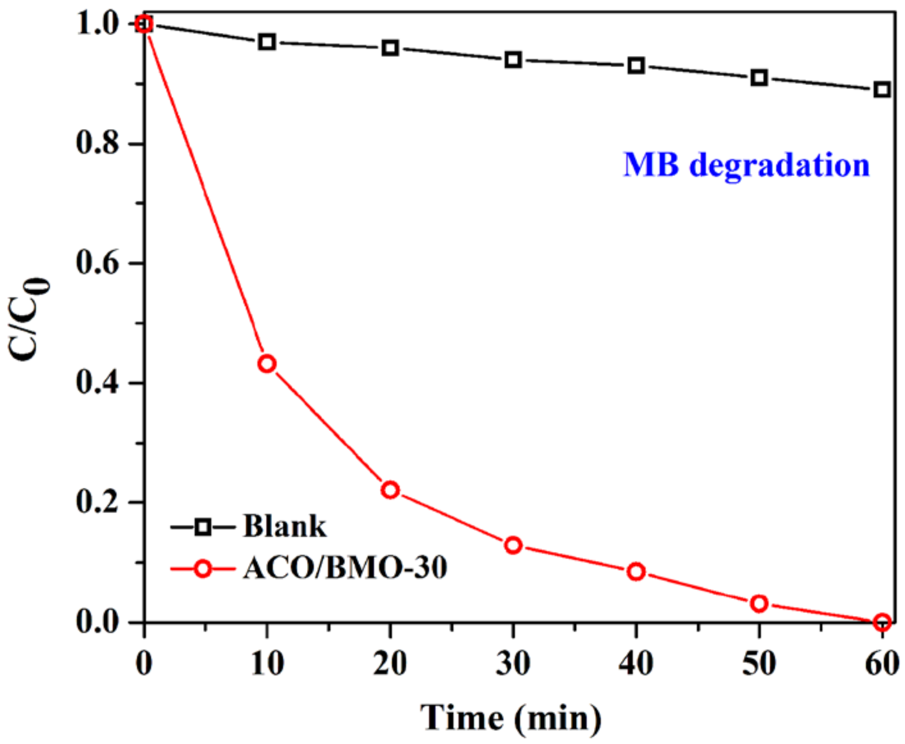

Figure S2: The photocatalytic degradation of MB over ACO/BMO-30.

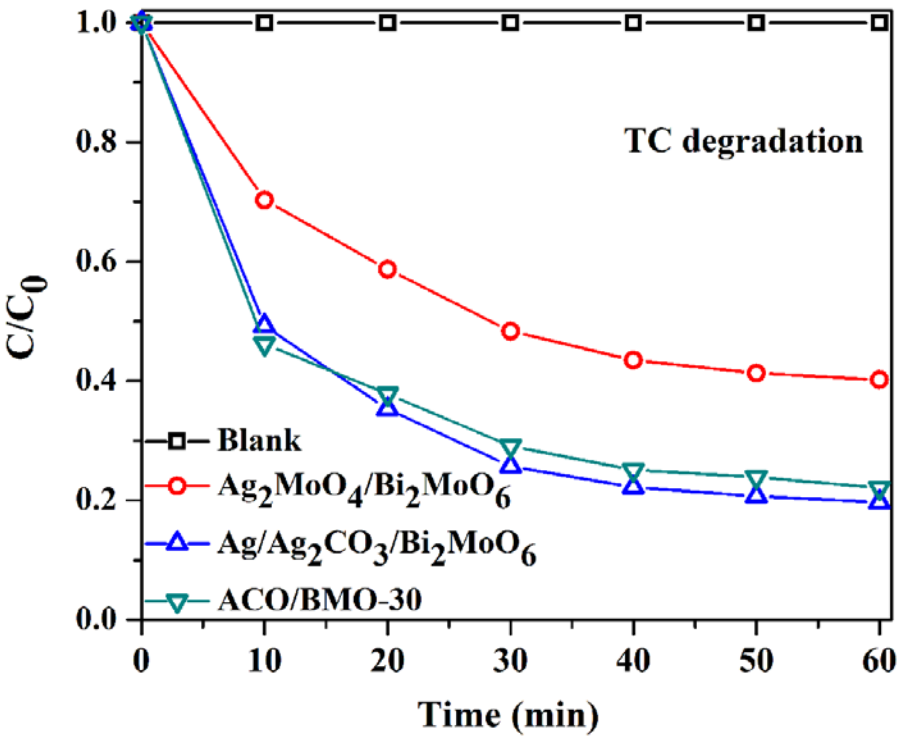

Figure S3: Photocatalytic degradation of TC (100 mL, 20 mg·L^{-1}) over ACO/BMO-30, $Ag/Ag_2CO_3/Bi_2MoO_6$, and Ag_2MoO_4/Bi_2MoO_6.

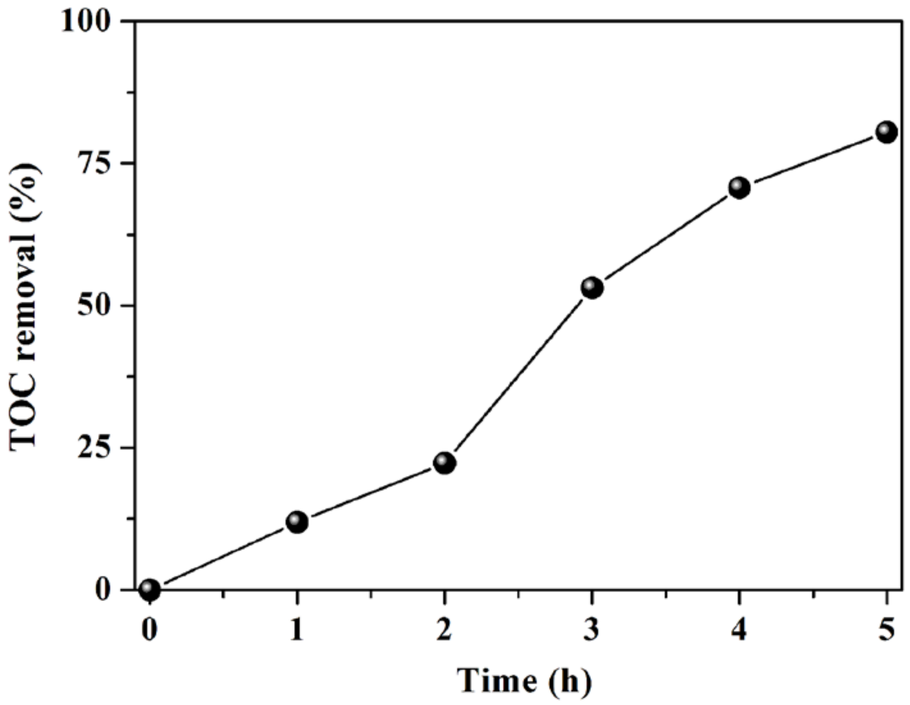

Figure S4: TOC removal during the photocatalytic reaction.

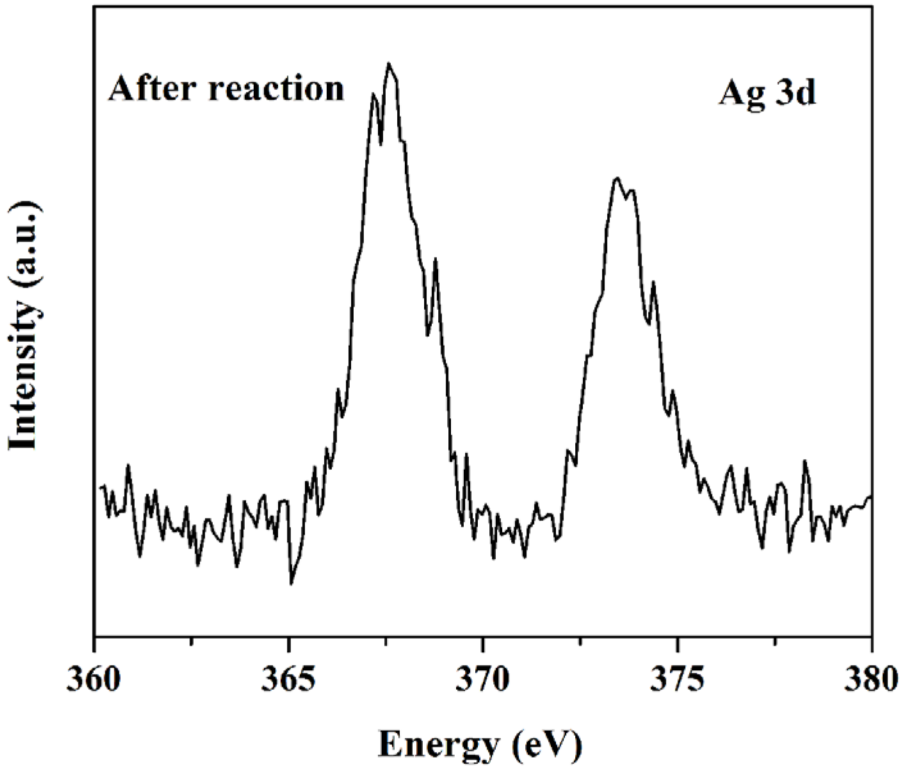

Figure S5: High-resolution XPS spectra of Ag 3d for ACO/BMO-30 after photocatalytic reaction.
